# Changes of Swimmers’ Emotional States during the Preparation of National Championship: Do Recovery-Stress States Matter?

**DOI:** 10.3389/fpsyg.2017.01043

**Published:** 2017-06-23

**Authors:** Philippe Vacher, Michel Nicolas, Guillaume Martinent, Laurent Mourot

**Affiliations:** ^1^Laboratory Psy-DREPI (EA 7458), University of Bourgogne Franche-ComtéDijon, France; ^2^Laboratory of Vulnerabilities and Innovation in Sport (EA 7428), University of Claude Bernard Lyon 1 – University of LyonVilleurbanne, France; ^3^Laboratory of Prognostic Markers and Regulatory Factors of Cardiovascular Diseases and Exercise Performance, Health, Innovation Platform (EA 3920), University of Bourgogne Franche-ComtéBesançon, France

**Keywords:** multilevel growth curve analyses, ecological conditions, emotions in sports, training, athletes

## Abstract

This study examined the trajectories of emotional states and their within-person synergies with perceived stress and recovery during a 4-month training period preceding the French swimming championships. A Multilevel Growth Curve Analysis approach was used with 16 high level swimmers. Five waves of assessments of emotional states, perceived stress and recovery were completed. Results indicated that emotional states were characterized by distinct trajectories during the training period preceding a major competition. Specifically, significant positive linear effects of time (i.e., linear increase over time) and negative quadratic effects of squared time (i.e., inverted U shape over time) on anxiety, dejection and anger were observed, whereas the opposite pattern of results was found for happiness and excitement. Moreover, level 2 perceived stress and recovery (i.e., inter-individual predictors) were significantly associated with athletes’ unpleasant and pleasant emotional states respectively. At level 1, perceived recovery (i.e., intra-individual predictor) was positively associated with happiness and excitement and negatively related to anxiety, dejection and anger. Finally, within-person interactions of general stress and recovery with time and squared time reached significance for excitement, whereas within-person interactions of specific and total stress with time and squared time reached significance for anxiety. Overall, this study provided insights into the central role played by perceived stress and recovery on the emotional states experienced by high level swimmers. Operational strategies were suggested in order to optimize the stress-recovery balance and in turn the athletes’ emotional states during a complete training program.

## Introduction

High level swimming requires intensive work and abnegation in order to perform and reach goals. However, high intensity training may endanger the stress-recovery balance and thus expose swimmers to deleterious psychological states such as overtraining, burnout or emotional disturbances (Lonsdale et al., 2009; Difiori et al., 2014). Such impaired states can be considered as a failure on the part of both athletes and coaches to accurately manage the training process. However, in high level swimming, distinguishing appropriate adaptation to training load (TL) from early maladaptation is quite difficult during periods of heavy training (e.g., stress-recovery unbalance, increase in unpleasant emotions, increase in cardiac response to submaximal exercise) (Schwellnus et al., 2016; Soligard et al., 2016). It is therefore a key issue for coaches and athletes to better understand the psychological process (e.g., emotional states) which takes place during the training period preceding major competitions (Meeusen et al., 2013).

In the perspective of preventing maladaptation, one of the most recent and relevant psychological tools which investigate both stress (nature and frequency) and recovery (behaviors) is the Recovery-Stress Questionnaire for Athletes (RESTQ-Sport; (Kellmann and Kallus, 2001). Using a biopsychological perspective as a theoretical background, several scholars have suggested that athletes have to manage their stress-recovery balance in order to perform optimally (e.g., Kellmann and Kallus, 2001; Coutts et al., 2007). Within this biopsychological framework (Kellmann and Kallus, 2001), recovery and stress states are treated using a multilevel approach, taking into consideration psychological, emotional, cognitive, behavioral/performance, and social aspects of stress and recovery. More particularly, the process of recovery cannot take place solely through the elimination of stress, rather it includes an action-oriented component (self-initiated activities, pro-active recovery) designed to optimize the situational conditions (reestablishment of psychological and physical strength). In this line of reasoning, under-recovery is considered as a precursor of maladaptation (Kellmann, 2002). Numerous studies using the RESTQ-Sport reported a dose-response relationship between training and stress-recovery states (Coutts et al., 2007; Filaire et al., 2013; Freitas et al., 2014). Indeed, stress states were positively associated, while recovery states were negatively associated with TL. It is worth noting that a hierarchical structure of the RESTQ-Sport has been proposed, leading researchers to mainly use second-order factors of general, sport-specific and/or total stress and recovery (Kellmann, 2010). In particular, a bulk of studies provided strong evidence that general, sport-specific and total stress and recovery scores could be used by researchers and practitioners interested in global measures of recovery and stress as they simultaneously encompass the physical, emotional, behavioral, and social processes postulated within the biopsychological framework (e.g., Kellmann and Kallus, 2001; Kellmann, 2010; Martinent et al., 2014; Nicolas et al., 2016). For instance, recent confirmatory factor analytic studies showed that: (a) the hierarchical structure of RESTQ-Sport provided an acceptable fit to the data; (b) internal consistency coefficients, item analysis as well as various reliability indicators provided evidence for the reliability of second-order RESTQ-Sport scores; and (c) relationships between second-order RESTQ-Sport scores, psychological (e.g., sport motivation, athlete burnout), physiological (e.g., heart rate variability) and objective (e.g., training load) theoretically relevant variables provided strong evidence for the convergent and discriminant validities of second-order RESTQ-Sport scores (Martinent et al., 2014; Nicolas et al., 2016).

In addition to the recovery-stress states, it has been previously shown that emotional states are also particularly affected by periods of intensive and/or reduced training among endurance athletes like swimmers (Morgan et al., 1988; O’Connor et al., 1989). However, such investigations have been conducted to a large extent using the Profile of Mood States (POMS), which was first developed for use with a clinical population and clearly centers on negative moods (five unpleasant emotions versus one pleasant emotion; (Jones et al., 2005). It has also been underlined that certain dimensions (e.g., fatigue or confusion) evaluated with the POMS are neither emotions, nor moods (Jones et al., 2005). Actually, “prototypical emotional episode” (i.e., commonly called emotion; Russell and Barrett, 1999; Russell, 2009), is characterized by antecedent event, affective quality (i.e., attributed to the antecedent event), core affect, attribution (i.e., core affect is attributed to the antecedent which then become the object), cognitive appraisal (i.e., by which the object is evaluated as relevant), instrumental actions (i.e., actions directed at the object), physiological and expressive changes, subjective conscious experiences (e.g., sense of urgency, indecision, confusion), emotional meta-experience (i.e., categorization of one’s state) and emotional regulation (i.e., by categorizing oneself, ones will place his current state and situation in a broader body knowledge). In this line of reasoning, it has been underlined that the cognitive appraisal which takes place in the “individual-object” transaction is a key element (Ekkekakis, 2013). It has also been argued that there is a unity between stress and emotions in the way that “*when there is stress there are also emotions and the reverse, although not always the case, often applies*” (Lazarus, 2006, p. 35). Based on this interdependence, it is clear that stress and emotions have large communalities in the way these embodied states of mind are aroused, coped with, and affect psychological well-being, functioning, and somatic health (Lazarus, 2006). Furthermore, by a methodological point of view, recommendations have been recently done concerning the fact that understanding stress-emotions psychological process involve to use longitudinal measures methods, or at least repeated measures research designs rather than cross-sectional ones (Lazarus, 2006).

In order to investigate a specific set of emotional states (Ekkekakis, 2013), and grounded within cognitive motivational relational framework of Lazarus’ (2000, 2006), we chose to focus our attention on the Sport Emotions Questionnaire (SEQ; Jones et al., 2005) which covers a large array of emotions experienced by athletes. This tool is of a special interest in the sense that it has been constructed in order to specifically measure emotion rather than mood or affect (Jones et al., 2005). Specifically, the authors retained three unpleasant emotions (anger, anxiety and dejection) and two pleasant emotions (happiness and excitement) in order to investigate the emotions experienced in sport settings (Jones et al., 2005; Arnold and Fletcher, 2015). As a matter of fact, taking into account positive emotions is of major importance in the understanding of the recovery process. Indeed, it has been repeatedly shown that observing a drop in negative mood states is insufficient in providing a comprehension of the recovery process (see Lundqvist and Kenttä, 2010). Moreover, based on the broaden-and-build theory (Fredrickson, 1998, 2001), positive emotions are of key importance because they broaden the scope of attention and thought-action repertoires. Along these lines, it has been shown that pleasant emotions induce coordinated changes in people’s thoughts, actions, and physiological responses which have long-lasting consequences (Fredrickson and Branigan, 2005). Such characteristics represent adaptive functions that are important for the recovery process and in a pro-active and long term perspective (Lundqvist and Kenttä, 2010), highlighting the necessity to investigate pleasant emotions in the prevention of impaired states in athletes. Finally, by taking into account a large range of pleasant and unpleasant emotions, Jones’ perspective respond to the literature recommendations and is of a particular interest in order to investigate the stress and emotions process (Lazarus, 2000).

To date, numerous studies have tried to monitor training load impact on psychological states (Coutts et al., 2007; Filaire et al., 2009; Nicolas et al., 2011; Schmitt et al., 2013; Freitas et al., 2014). Along these lines, the existence of a relationship between training volume and mood disturbances was demonstrated several decades ago (Raglin, 1993). Increases in training volume are mirrored in emotional disturbances (more anger, depression and tension, and less vigor), while volume reductions (e.g., tapering periods) are mirrored in emotional improvements (see Morgan et al., 1987a,b; Raglin et al., 1991; Kellmann and Kallus, 2001; Millet et al., 2005). In this line of reasoning, investigations must be conducted in respect to training loads variations and in coherence with the physical preparation training plan. Indeed, training periodization include variations in training load which correspond to a specific stressor (Foster, 1998; Winsley and Matos, 2010). These moments in which training load vary may impact ones’ appraisals variations and fit with changes in athletes’ emotional experiences.

Because emotions serve many functions linked to the training process (e.g., physiological responses, elicitation of the flexibility of behavioral responses or motivational impact; see Rolls, 2013), and TL impacts both emotions and recovery states, it is a key issue for coaches and athletes to increase their understanding of relationships between emotions and stress-recovery states in order to better anticipate impaired states due to training. Despite the fact that a rich literature has investigated stress-recovery states and athletes’ emotional states during the training process, we failed to find a study concerning the effects of stress-recovery states on longitudinal trajectories of emotional states within an ecological training program in athletes preparing a major competition. Previous studies showed that stress is accompanied by emotional symptoms like anxiety and anger (Lazarus, 1993; Kellmann and Günther, 2000). At the same time, emotional exhaustion is positively associated with higher level in stress states and lower level in recovery states (Maslach et al., 1997; Kellmann and Kallus, 2001). In view of the literature mentioned above, it could be expected that unpleasant emotions would increase during building up periods of training and then decrease during tapering periods, with opposite patterns for pleasant emotions. Such expectations implied to explore the linear and the quadratic trajectories of stress, recovery and emotional states all around the training periodization. Moreover, because of the links between emotional states and stress-recovery states (Kellmann and Günther, 2000), it could be fruitful to investigate the effects of stress-recovery states on emotional trajectories. In particular, it could be useful to explore whether the linear and/or quadratic increases or decreases in a specific athlete’s emotional states might be accelerated or decelerated at times when the score of the athlete perceived stress (or recovery) was higher (or lower) than his own average (i.e., within-person synergies of emotional states with perceived stress and recovery). As a whole, more studies are needed concerning the trajectories of emotional states and the within-person synergies of emotional states with other psychological process (e.g., perceived stress and recovery), especially during training periods preceding important competitions. Such studies will enable all actors to progress beyond the stage of “athletes’ states,” toward the opening up of a pro-active and global management of stress, recovery and emotional states.

In this perspective, the present study sought to explore how emotional states (i.e., happiness, excitement, anxiety, dejection and anger) evolved over a 4-month training period preceding the French swimming championships. More specifically, we examined the linear and quadratic trajectories of anxiety, dejection, anger, excitement and happiness over a 4-month training period preceding the target competition of the season (i.e., the French swimming championships). In addition, we also explored the effects of perceived stress and recovery on the athletes’ emotional states experienced by swimmers during the training period preceding an important competition. In line with past research (Morgan et al., 1987b; Raglin et al., 1991; Millet et al., 2005; Martinent et al., 2014), we hypothesized that unpleasant emotional states (i.e., anxiety, dejection and anger) would increase at first and then decrease during the tapering period (i.e., significant quadratic slopes) whereas pleasant emotional states (excitement, happiness) would show the opposite pattern of results. In light of the aforementioned theoretical rationale and empirical evidence, we also hypothesized that perceived stress would be associated with high levels of unpleasant emotional states, whereas perceived recovery would be associated with high levels of pleasant emotional states.

## Materials and Methods

### Participants

Sixteen high level swimmers (10 males and 6 females; *M*age = 18.19; *SD* = 1.47) voluntarily participated in the study. On average, they trained 20.45 h per week (*SD* = 2.35), had been competing in their sport for an average of 8.19 years (*SD* = 1.47) and at a national level for at least 2 years (*M* = 4.19; *SD* = 0.98) before the study. The swimmers’ coaches were contacted to obtain permission to approach their athletes about participation in the study. Participants signed an informed consent form approved by the local ethics committee approval and by the swimming club’s executive committee (as well as from the parents of underage athletes). *A priori* power analysis was performed using Power IN Two-level designs software which is designed to estimate standard errors of regression coefficients in hierarchical linear models for power calculations (Snijders and Bosker, 1993). If α is chosen at 0.05, a medium effect size of 0.50 is what we expect and a sample of 16 participants along five measurement points is chosen, then we can derive that power is 0.87 for the present study. As a rule of thumb, Cohen (1988) suggests that power is high when it is at least 0.80. Thus, result of power analysis provided evidence that sample size of the present study is acceptable.

### Measures

#### Sport Emotion Questionnaire

A French translation of the Sport Emotion Questionnaire (SEQ, Jones et al., 2005) was used to assess emotional states experienced by athletes during the training process. The SEQ is a 22-item instrument assessing the intensity of five emotional states (anxiety, dejection, anger, excitement and sadness) on a scale ranging from 0 (*not at all*) to 4 (*extremely*). The SEQ demonstrates acceptable face, content, factorial and concurrent validity (Jones et al., 2005). Translation of the SEQ into French was conducted according to a standardized back-translation procedure. The SEQ was first translated into French and sent to two bilingual translators who then translated it back into English. Differences were then discussed and resolved so that the original meaning of each original item was considered to be expressed in the final French version. Cronbach’s alphas ranged from 0.70 to 0.92 (see **Table 1**).

**Table 1:** Descriptive statistics for the five waves.

	Time 1		Time 2		Time 3		Time 4		Time 5	
	*M (SD)* Range	α	*M (SD)* Range	α	*M (SD)* Range	α	*M (SD)* Range	α	*M (SD)* Range	α
Anxiety	1.86 (0.83)	0.90	2.00 (0.72)	0.77	2.44 (0.94)	0.82	2.30 (1.12)	0.88	1.95 (0.60)	0.70
Dejection	1.39 (0.42)	0.71	1.65 (0.72)	0.84	2.00 (0.92)	0.89	1.98 (0.84)	0.85	1.45 (0.52)	0.86
Anger	1.50 (0.66)	0.88	1.77 (0.81)	0.85	1.92 (0.72)	0.81	1.77 (0.75)	0.79	1.52 (0.55)	0.81
Excitement	3.69 (1.00)	0.80	3.50 (0.88)	0.77	3.02 (1.00)	0.84	3.08 (1.05)	0.90	3.48 (0.90)	0.70
Happiness	3.95 (0.83)	0.79	3.75 (0.82)	0.78	3.13 (1.05)	0.89	3.36 (1.05)	0.92	3.75 (0.85)	0.84
General stress	2.00 (0.60)	0.89	2.76 (0.59)	0.94	3.20 (0.70)	0.82	3.10 (0.79)	0.86	2.19 (0.77)	0.95
Sport-specific stress	2.09 (0.67)	0.87	2.92 (0.66)	0.93	3.33 (0.85)	0.88	3.20 (0.79)	0.90	2.28 (0.59)	0.94
Total stress	2.05 (0.59)	0.92	2.84 (0.58)	0.96	3.26 (0.75)	0.90	3.15 (0.77)	0.92	2.24 (0.67)	0.94
General recovery	4.11 (0.68)	0.87	3.69 (0.78)	0.97	3.38 (0.68)	0.87	3.69 (0.77)	0.94	3.97 (0.74)	0.98
Sport-specific recovery	3.15 (0.71)	0.70	3.06 (0.75)	0.93	2.67 (0.71)	0.82	3.00 (0.71)	0.88	3.57 (0.89)	0.96
Total recovery	3.63 (0.64)	0.88	3.37 (0.73)	0.97	3.03 (0.64)	0.88	3.35 (0.70)	0.94	3.77 (0.76)	0.97

*Time 1 corresponded to 1-week of resumed training characterized by a low training load (TL) (internal TL; *M* = 1939, *SD* = 1120 a.u. External TL; *M* = 19, *SD* = 5 km). T2 to T4 corresponded to three development periods of 3-weeks (3^∗^3 weeks) characterized by a progressive increase in internal TL (T2; *M* = 4990, *SD* = 1195 a.u, T3; *M* = 6763, *SD* = 1273 a.u, T4; *M* = 7335, *SD* = 1017 a.u) and external training load (T2; *M* = 41, SD 10 km, T3; *SD* = 50, *M* = 9 km, T4; *M* = 57, *SD* = 7 km). T5 corresponded to a tapering period of 2.5-weeks characterized by an important reduction in internal (*M* = 1472, *SD* = 273 a.u) and external (*M* = 21, *SD* = 4 km) TL. Cronbach’s alpha (α), Mean (M) and Standard Deviation (SD) are presented for each time and emotion*.

#### Recovery-Stress Questionnaire for Athletes

The short French version of the Recovery-Stress Questionnaire for Athletes was used to measure the recovery-stress state of the swimmers (Nicolas et al., 2016). Like the original version of 76 items (Kellmann and Kallus, 2001), the short version (RESTQ-36-R-Sport; Kellmann and Kallus, 2016) measures the extent to which an individual is physically and/or mentally stressed, and whether or not the person is capable of using individual strategies for recovery both in a general and a sport-specific context. The RESTQ-36-R-Sport comprises 12 three-item subscales including six general subscales concerning stress (general stress, social stress, and fatigue) or recovery (somatic relaxation, general well-being, and sleep quality). It also includes six specific subscales which address the sport dimension of stress (disturbed breaks, emotional exhaustion and fitness/injury) and recovery (fitness/being in shape, personal accomplishment, and self-efficacy) processes from a physical, emotional, behavioral and social perspective (see Nicolas et al., 2016). Recent research has supported the validity and reliability of the French RESTQ-36-R-Sport which provides a less cumbersome and better fitting model of athletes’ perceptions of stress and recovery states (Nicolas et al., 2016). Based on the biopsychological theoretical perspective (Kellmann, 2010) and consistent with previous RESTQ-Sport studies (e.g., Kellmann and Kallus, 2001; Martinent et al., 2014; Nicolas et al., 2016), we used the General, Specific and Total scores of Stress and Recovery (i.e., respectively GS, GR, SS, SR, TS and TR) in order to adopt a holistic perspective of the athletes’ recovery and stress states. The response scale asked participants to rate the frequency of each item over the preceding 3 days/nights on a scale of 0 (*never*) to 6 (*always*). Cronbach’s alphas ranged from 0.82 to 0.95 (see **Table 1**).

### Procedure

Data collection occurred at five time points during a 4-month training period preceding the French swimming championships (i.e., the target competition of the season). External training load was assessed by the total number of kilometers of the weekly sessions. Internal training load was assessed by multiplying the athlete’s “rating of perceived exertion” (RPE, on a 1–10 scale) obtained 30 min after the completion of the training session by the duration (in minutes) of the session (Foster et al., 2001; Wallace et al., 2009). By totaling all RPE sessions over the week, we obtained the weekly training load of each swimmer (Meeusen et al., 2013), expressed in arbitrary units (a.u). This method has demonstrated its relevance in quantifying TL in numerous sports (Impellizzeri et al., 2004; Haddad et al., 2011; Lovell et al., 2013; Scott et al., 2013) including swimming (Wallace et al., 2009). The five waves were selected because they represented key time points in the training process: the first (T1) corresponded to 1-week of resumed training characterized by a low internal (*M* = 1939, *SD* = 1120 a.u) and external TL (*M* = 19, *SD* = 5 km). Following T1, coaches planned three development periods of 3-weeks (3^∗^3 weeks) characterized by a progressive increase in internal TL (T2; *M* = 4990, *SD* = 1195 a.u, T3; *M* = 6763, *SD* = 1273 a.u, T4; *M* = 7335, *SD* = 1017 a.u) and external TL (T2; *M* = 41, *SD* = 10 km, T3; *M* = 50, *SD* = 9 km, T4; *M* = 57, *SD* = 7 km). After T4, a tapering period (T5) of 2.5-weeks was included in the training periodization in order to bring athletes up to the target competition, which began the day after the final measures. The T5 period was characterized by an important reduction in internal (*M* = 1472, *SD* = 273 a.u) and external (*M* = 21, *SD* = 4 km) training loads.

### Data Analyses

Multilevel growth curve analyses (MGCAs) were used to examine the trajectories of athletes’ emotional states (Singer and Willett, 2003). All analyses were conducted using the R package labeled lme4 (Bates et al., 2015). Separate analyses were conducted for each of the five distinct emotional states (i.e., anxiety, dejection, anger, excitement and happiness). Multilevel models extend multiple regressions to nested data (i.e., data that are hierarchically structured). Specifically, repeated measurements (Level 1 units of analysis) were nested within individuals (Level 2 units of analysis) because several observations were gathered for each individual. Multiple regression models are based on the assumption that all observations are independent, which may not be the case with nested data. Hence, multilevel models are a flexible approach that can be applied to evaluate inter-individual differences in intra-individual changes over time (i.e., each participant has his own curve). Thus, by taking into account the hierarchical structure of the data, multilevel models provide unbiased estimates of the parameters (Singer and Willett, 2003). Moreover, because collinearity of predictors can unduly influence results of MGCAs in potentially unfavorable ways, the data set was screened for collinearity. No collinearity was detected.

Firstly, a two-level model estimated both the average and the individual differences in growth. Specifically, at Level 1, time (linear trajectory) and squared time (quadratic trajectory) were entered as predictors to estimate the average intercept (β*0*), the average linear growth (β*1*) and the average quadratic growth (β*2*). The time variable was centered on the first wave. Thus, the intercept should be interpreted as the level of athletes’ emotional states at the start of the training period for the preparation of the French swimming championships (i.e., 1-week resumed training). The random effects of the intercept, linear slope, and quadratic slope were included in each of the models. Whereas initial MGCA models included both the linear and quadratic functions of emotional trajectories, it must be noted that the MGCA models with both the linear and quadratic functions were then compared to their respective MGCA models with only the linear function. Thus, the quadratic parameters were included in the subsequent MGCA models only if chi-square tests provided evidence of a significant improvement of fit (Doron and Martinent, 2015).

Secondly, the final MGCA models estimated the within-person (as Level 1 predictors with group mean centring) and the between-person (as Level 2 predictors with grand mean centring) main effects of perceived stress and recovery on the dependent variables (i.e., emotional states) as well as the within-level moderating effects of perceived stress and recovery in the linear (i.e., stress^∗^time and recovery^∗^time) and quadratic trajectories (i.e., stress^∗^squared time and recovery^∗^squared time) of the dependent variables. It is noteworthy that group mean centering was used for all Level 1 predictors based on the rationale that grand-mean centering or no centering may produce biased point estimates (Zhang et al., 2009). It is noteworthy that three series of MGCA models were computed in which the general (i.e., model 2A), sport-specific (i.e., model 2B) and total (i.e., model 2C) stress and recovery scores of the RESTQ-36-R-Sport were included as predictors of emotional states in the MGCA models.

## Results

Before proceeding to test the hypotheses, we analyzed the systematic within- and between-individual variance in emotional states. The results of the null models (see **Table 2**) indicated that there was substantial within- and between-individual variance for all the emotional states: σ^2^ (i.e., variance in level-1 residual) ranged from 0.24 to 0.53 whereas τ_00_ (i.e., variance in level-2 residual) ranged from 0.17 to 0.45. The intraclass correlations [ICC = τ_00_/(σ^2^ + τ_00_)] ranged from 0.32 to 0.51, indicating that between-individual variance accounts for 32–51% percent of the total variance in the emotional states. This would suggest that between 49 and 68% of the overall variance (both systematic and error) is attributable to within-individual variation, suggesting that emotional states vary considerably.

**Table 2:** Null models of the emotional states.

	Anxiety	Dejection	Anger	Excitement	Happiness
*Fixed effects - Estimates (Standard errors)*					
Intercept	2.11^∗∗∗^ (0.16)	1.69^∗∗∗^ (0.12)	1.68^∗∗∗^ (0.14)	3.37^∗∗∗^ (0.19)	3.60^∗∗∗^ (0.17)
*Random effects - Variance (Standard deviation)*					
Intercept	0.33 (0.58)	0.17 (0.41)	0.25 (0.50)	0.45 (0.67)	0.36 (0.60)
Residual	0.40 (0.63)	0.36 (0.60)	0.24 (0.49)	0.50 (0.70)	0.53 (0.73)
-2^∗^loglikelihood	176.1	161.6	138.8	193.8	194.9
Intra class correlations	0.45	0.32	0.51	0.47	0.40

*^∗∗∗^p <0.001*.

### Development of Emotional States during the Training Process (Model 1)

Multilevel growth curve analyze models with both the linear and quadratic functions of emotional trajectories were then compared to their respective MGCA models with only the linear function of emotional trajectories. Results of chi-square tests provided evidence of a significant improvement of fit when quadratic parameters were included in the MGCA models for each of the emotional states. Hence, quadratic parameters were included in the MGCA model of each of the emotional states. Results revealed a significant positive linear effect of time on anxiety (β = 0.49), dejection (β = 0.60) and anger (β = 0.40) as well as a significant negative linear effect of time on happiness (β = -0.62) and excitement (β = -0.69) (see **Table 3** for more details). As expected from the descriptive statistics presented in **Table 1**, the results of MGCA model 1 also revealed a significant quadratic effect of time on anxiety (β = -0.11), dejection (β = -0.14) and anger (β = -0.10) as well as a significant positive quadratic effect of time on happiness (β = 0.14) and excitement (β = 0.16) (see **Table 3** for more details). Thus, happiness and excitement decreased at first and then increased during the tapering period whereas the opposite pattern of development was observed for anxiety, dejection and anger.

**Table 3:** Growth curve models of emotional states during the training process.

	Anxiety	Dejection	Anger	Excitement	Happiness
*Fixed effects - Estimates (Standard errors)*					
Intercept	1.79^∗∗∗^ (0.18)	1.32^∗∗∗^ (0.11)	1.49^∗∗∗^ (0.17)	3.78^∗∗∗^ (0.24)	4.04^∗∗∗^ (0.19)
Time	0.49^∗∗^ (0.17)	0.60^∗∗^ (0.19)	0.40^∗∗^ (0.14)	-0.62^∗∗^ (0.21)	-0.69^∗∗^ (0.22)
Time^2^	-0.11^∗∗^ (0.04)	-0.14^∗∗^ (0.04)	-0.10^∗∗^ (0.03)	0.14^∗∗^ (0.05)	0.16^∗∗^ (0.05)
*Random effects - Variance (Standard deviation)*					
Intercept	0.22 (0.47)	0.01 (0.10)	0.28 (0.53)	0.62 (0.78)	0.25 (0.50)
Time	0.05 (0.22)	0.31 (0.56)	0.09 (0.31)	0.24 (0.49)	0.34 (0.58)
Time^2^	0.00 (0.05)	0.01 (0.12)	0.00 (0.06)	0.01 (0.09)	0.01 (0.11)
Residual	0.34 (0.58)	0.22 (0.47)	0.18 (0.42)	0.35 (0.59)	0.34 (0.59)
-2^∗^loglikelihood	166.4	134.3	126.9	181.2	176.8

*^∗∗∗^p <0.001, *^∗∗^p <*0.01, ^∗^*p <*0.05*.

### Within-Person Synergies of Emotional States, Perceived Stress and Recovery (Models 2A, 2B, and 2C)

Concerning the between-person main effects of perceived stress and recovery (i.e., Level 2 predictors), general stress was significantly associated with dejection (β = 0.72), anger (β = 1.07) and excitement (β = -0.65) whereas general recovery was significantly associated with anxiety (β = -0.47), excitement (β = 0.57) and happiness (β = 0.59) (see **Table 4**). Similarly, sport-specific and total stress were positively associated with anxiety (β = 1.11 and 1.20 respectively), dejection (β = 0.77 and 0.89), and anger (β = 1.09 and 1.22), whereas sport-specific and total recovery were positively associated with excitement (β = 1.09 and 1.04) and happiness (β = 0.53 and 0.65) (see **Tables 5**, **6**).

**Table 4:** Effects of general stress and recovery on the growth curve models of emotional states. Model 2A.

	Anxiety	Dejection	Anger	Excitement	Happiness
*Fixed effects - Estimates (Standard errors)*					
Intercept	2.12^∗∗∗^ (0.20)	1.59^∗∗∗^ (0.14)	1.81^∗∗∗^ (0.18)	3.41^∗∗∗^ (0.22)	3.77^∗∗∗^ (0.19)
Time	-0.15 (0.20)	0.08 (0.20)	-0.00 (0.17)	0.19 (0.20)	0.06 (0.22)
Time^2^	0.03 (0.05)	-0.02 (0.05)	-0.02 (0.04)	-0.06 (0.05)	-0.03 (0.05)
General stress (GS)	0.01 (0.23)	-0.00 (0.18)	0.14 (0.21)	-0.09 (0.25)	-0.00 (0.24)
General recovery (GR)	-0.85^∗^ (0.39)	-0.71^∗^ (0.28)	-0.71^∗^ (0.34)	0.69*^¥^* (0.41)	0.57 (0.38)
GS ^∗^ Time	0.59 (0.37)	0.36 (0.29)	0.31 (0.30)	-0.92^∗∗^ (0.34)	-0.44 (0.29)
GR ^∗^ Time	0.42 (0.58)	0.29 (0.44)	0.83 (0.49)	-1.03^∗^ (0.51)	0.20 (0.48)
GS ^∗^ Time^2^	-0.14 (0.10)	-0.07 (0.08)	-0.09 (0.08)	0.24^∗∗^ (0.09)	0.10 (0.08)
GR ^∗^ Time^2^	-0.08 (0.15)	-0.02 (0.12)	-0.18 (0.13)	0.33^∗^ (0.14)	-0.02 (0.12)
GS Level 2	0.50 (0.33)	0.72^∗∗^ (0.21)	1.07^∗∗∗^ (0.29)	-0.65^∗^ (0.31)	-0.50*^¥^* (0.28)
GR Level 2	-0.47^∗^ (0.24)	0.00 (0.14)	0.03 (0.20)	0.57^∗∗^ (0.22)	0.59^∗∗^ (0.20)
*Random effects - Variance (Standard deviation)*					
Intercept	0.17 (0.41)	0.01 (0.10)	0.21 (0.46)	0.33 (0.57)	0.23 (0.48)
Time	0.00 (0.03)	0.24 (0.49)	0.03 (0.17)	0.10 (0.32)	0.32 (0.57)
Time^2^	0.00 (0.00)	0.01 (0.11)	0.00 (0.03)	0.00 (0.05)	0.01 (0.11)
Residual	0.24 (0.49)	0.16 (0.39)	0.16 (0.39)	0.18 (0.42)	0.14 (0.37)
-2^∗^loglikelihood	129.0	98.8	101.1	120.1	108.6

*^∗∗∗^*p* < 0.001, ^∗∗^*p* < 0.01, ^∗^*p* < 0.05, ^¥^*p* = 0.09*.

**Table 5:** Effects of sport-specific stress and recovery on the growth curve models of emotional states. Model 2B.

	Anxiety	Dejection	Anger	Excitement	Happiness
*Fixed effects - Estimates (Standard errors)*					
Intercept	1.83^∗∗∗^ (0.16)	1.47^∗∗∗^ (0.11)	1.61^∗∗∗^ (0.16)	3.58^∗∗∗^ (0.21)	3.94^∗∗∗^ (0.20)
Time	0.01 (0.18)	0.11 (0.18)	0.15 (0.17)	0.01 (0.18)	-0.16 (0.22)
Time^2^	0.01 (0.04)	-0.02 (0.04)	-0.04 (0.04)	-0.03 (0.04)	0.02 (0.05)
Specific stress (SS)	-0.14 (0.15)	0.13 (0.12)	0.14 (0.15)	-0.10 (0.16)	0.04 (0.17)
Specific recovery (SR)	-0.93^∗∗^ (0.28)	-0.14 (0.21)	-0.24 (0.28)	0.90^∗∗^ (0.33)	1.00^∗∗^ (0.33)
SS ^∗^ Time	1.02^∗∗∗^ (0.30)	0.53^∗^ (0.27)	0.26 (0.29)	-0.20 (0.33)	-0.34 (0.31)
SR ^∗^ Time	0.68 (0.39)	0.13 (0.33)	0.34 (0.39)	-0.16 (0.44)	0.06 (0.41)
SS ^∗^ Time^2^	-0.23^∗∗^ (0.08)	-0.12 (0.07)	-0.07 (0.08)	0.03 (0.09)	0.05 (0.08)
SR ^∗^ Time^2^	-0.11 (0.09)	-0.04 (0.08)	-0.08 (0.09)	0.02 (0.10)	-0.08 (0.09)
SS Level 2	1.11^∗∗∗^ (0.29)	0.77^∗∗∗^ (0.22)	1.09^∗∗∗^ (0.26)	0.10 (0.26)	-0.26 (0.40)
SR Level 2	0.09 (0.19)	-0.00 (0.14)	-0.00 (0.17)	1.09^∗∗∗^ (0.17)	0.53^∗^ (0.26)
*Random effects - Variance (Standard deviation)*					
Intercept	0.11 (0.34)	0.00 (0.00)	0.15 (0.38)	0.35 (0.60)	0.32 (0.56)
Time	0.13 (0.36)	0.19 (0.43)	0.05 (0.21)	0.07 (0.26)	0.32 (0.56)
Time^2^	0.00 (0.06)	0.01 (0.09)	0.00 (0.04)	0.00 (0.04)	0.01 (0.11)
Residual	0.16 (0.40)	0.14 (0.37)	0.16 (0.40)	0.18 (0.42)	0.15 (0.39)
-2^∗^loglikelihood	116.6	95.6	105.0	122.1	128.7

*^∗∗∗^*p* < 0.001, ^∗∗^*p* < 0.01, ^∗^*p* < 0.05*.

**Table 6:** Effects of total stress and recovery on the growth curve models of emotional states. Model 2C.

	Anxiety	Dejection	Anger	Excitement	Happiness
*Fixed effects - Estimates (Standard errors)*					
Intercept	2.01^∗∗∗^ (0.16)	1.56^∗∗∗^ (0.13)	1.72^∗∗∗^ (0.17)	3.47^∗∗∗^ (0.21)	3.80^∗∗∗^ (0.19)
Time	-0.11 (0.18)	0.05 (0.19)	0.04 (0.17)	0.16 (0.19)	0.02 (0.21)
Time^2^	0.03 (0.04)	-0.01 (0.04)	-0.02 (0.04)	-0.06 (0.04)	-0.02 (0.05)
Total stress (TS)	-0.13 (0.17)	0.14 (0.15)	0.16 (0.17)	-0.03 (0.20)	0.06 (0.19)
Total recovery (TR)	-1.29^∗∗∗^ (0.34)	-0.51*^¥^* (0.27)	-0.70^∗^ (0.35)	1.04^∗∗^ (0.39)	1.07^∗∗^ (0.38)
TS ^∗^ Time	0.99^∗^ (0.38)	0.52 (0.32)	0.38 (0.34)	-0.55 (0.37)	-0.25 (0.34)
TR ^∗^ Time	1.02*^¥^* (0.53)	0.44 (0.44)	0.89 (0.50)	-0.61 (0.55)	0.23 (0.50)
TS ^∗^ Time^2^	-0.24^∗^ (0.10)	-0.12 (0.09)	-0.11 (0.09)	0.13 (0.10)	0.03 (0.09)
TR ^∗^ Time^2^	-0.21 (0.13)	-0.10 (0.11)	-0.20 (0.12)	0.16 (0.13)	-0.10 (0.12)
TS Level 2	1.20^∗∗∗^ (0.32)	0.89^∗∗∗^ (0.22)	1.22^∗∗∗^ (0.28)	-0.08 (0.29)	-0.38 (0.37)
TR Level 2	0.02 (0.22)	0.05 (0.15)	0.06 (0.19)	1.04^∗∗∗^ (0.20)	0.65^∗^ (0.25)
*Random effects - Variance (Standard deviation)*					
Intercept	0.08 (0.28)	0.01 (0.12)	0.19 (0.44)	0.32 (0.56)	0.26 (0.51)
Time	0.05 (0.22)	0.21 (0.46)	0.04 (0.21)	0.07 (0.27)	0.25 (0.50)
Time^2^	0.00 (0.03)	0.01 (0.09)	0.00 (0.04)	0.00 (0.03)	0.01 (0.10)
Residual	0.18 (0.42)	0.14 (0.37)	0.15 (0.39)	0.17 (0.41)	0.14 (0.37)
-2^∗^loglikelihood	118.7	93.5	99.1	116.7	114.8

*^∗∗∗^*p* < 0.001, ^∗∗^*p <*0.01, ^∗^*p* < 0.05, ^¥^*p* = 0.06*.

Concerning the within-person main effects of perceived stress and recovery (i.e., Level 1 predictors), general recovery was negatively related to anxiety (β = -0.85), dejection (β = -0.71) and anger (β = -0.71) and marginally positively associated with excitement (β = 0.69, *p* = 0.09) (see **Table 4**). In other words, when athletes reported higher scores of perceived recovery (than their own averages), they also reported higher scores of excitement and lower scores of anxiety, dejection and anger. Similarly, sport-specific recovery was negatively related to anxiety (β = -0.93) and positively associated with excitement (β = 0.90) and happiness (β = 1.00) (see **Table 5**) whereas total recovery was negatively associated with anxiety (β = -1.29), dejection (β = -0.51, *p* = 0.06) and anger (β = -0.70) and positively related to excitement (β = 1.04) and happiness (β = 1.07) (see **Table 6**).

Within-person interactions of general stress and general recovery with time (β = -0.92 and -1.03 respectively) and with squared time (β = 0.24 and 0.33) (Level 1 predictors) reached significance in the case of excitement (see **Table 4**). The linear trajectory of excitement seems to decelerate at time points during which athletes also had higher levels (β = -0.73 and -0.84 respectively) compared to lower levels (β = 1.11 and 1.22) of general stress and recovery. In contrast, the quadratic trajectory of excitement seems to accelerate at time points during which athletes also had higher levels (β = 0.18 and 0.27) compared to lower levels (β = -0.30 and -0.39) of general stress and recovery. Otherwise, within-person interactions of sport-specific and total stress with time (β = 1.02 and 0.99 respectively) and with squared time (β = -0.23 and -0.24) reached significance for anxiety (see **Tables 5**, **6**). Therefore, the linear trajectory of anxiety accelerated at time points during which athletes also had higher levels (β = 1.03 and 0.88) compared to lower levels (β = -1.01 and -1.10) of sport-specific and total stress, whereas the quadratic trajectory of anxiety decelerated at time points during which athletes also had higher levels (β = -0.22 and -0.18) compared to lower levels (β = 0.24 and 0.27) of sport-specific and total stress.

## Discussion

This study explored the trajectories of a specific set of emotional states (i.e., happiness, excitement, anxiety, dejection and anger) during a 4-month training period preceding the French swimming championships (the target competition of the season). The influence of perceived stress and perceived recovery on the levels and trajectories of emotional states was also explored using a MGCA approach. By considering pleasant and unpleasant emotions measured by the SEQ (Jones et al., 2005), distinct trajectories for pleasant (i.e., happiness and excitement) and unpleasant (i.e., anxiety, dejection and anger) emotions were identified during the training periodization. Significant relationships were also revealed between recovery-stress and emotional states, indicating impaired psychological states in athletes. Thus, the findings suggested that the several emotional states experienced by the high-level swimmers during the training process were characterized by different dynamics and relationships with perceived stress and recovery within the 4-months preceding the French swimming championships.

In the context of the present study, the results of the MGCA model 1 showed that the quadratic effect of time is a significant predictor of all the swimmers’ emotional states (e.g., anxiety, dejection, anger, excitement and happiness). Hence, results systematically showed an increase in unpleasant emotions during the development periods, followed by a deceleration during the tapering period. In contrast, a decrease was noted in excitement and happiness during the development periods followed by an increase near the target competition of the season (i.e., tapering period). Based on Jones (1995), it is likely that during development periods, swimmers felt more unpleasant emotions, fewer pleasant ones and lower positive expectations of their ability to cope and reach goals. The tapering period which followed induced an increase in pleasant emotions associated with the ability to cope and reach goals (e.g., happiness and excitement).

It is also commonly accepted that major emotional disturbance coincides with an exhausting TL (see Millet et al., 2005; Meeusen et al., 2013), and that tapering periods result in a reduction in unpleasant emotions associated with an increase in vigor (a pleasant emotion) referred to as the iceberg profile (Morgan et al., 1987a,b; Raglin et al., 1991). Our results are consistent with the literature in demonstrating that stressful situations (i.e., development periods) induced a decrease in swimmers’ experience of happiness and excitement and were associated with increased anxiety, dejection and anger (Schiffrin and Nelson, 2010). Conversely, the increase in excitement and happiness observed during the tapering period could be understood as a positive adaptation to the reduction in both external and internal TL (Lane, 2007).

A central aspect of the present research, and one that has been overlooked in the sport literature was the documentation of the relationships between emotional states (i.e., happiness, excitement, anxiety, dejection and anger), perceived stress and perceived recovery during the training process leading to the target competition of the season. At a between-person level of analysis (i.e., Level 2 predictors), perceived stress and recovery were significantly associated with athletes’ unpleasant and pleasant emotional states respectively. Specifically, perceived stress positively predicted the levels (intercepts) of unpleasant emotions (anxiety, dejection and anger) experienced by swimmers whereas perceived recovery positively impacted their levels of pleasant emotions (i.e., excitement and happiness). At a within-person level of analysis (i.e., Level 1 predictors), results of the MGCAs highlighted the central role played by perceived recovery (and not perceived stress) in the emotions experienced by swimmers.

The fact that the recovery states measured by the RESTQ-36-R-Sport affect the level of pleasant and unpleasant emotional states may represent an important step forward, in that it provides practical information on a process which could place athletes in (non)functional dynamics. Indeed, a large part of the recovery-stress states literature investigates time-variations and associations between recovery-stress balance and psycho-physiological markers (e.g., cortisol, alpha amylase, mood states, emotions, training load; Coutts et al., 2007; Brink et al., 2012; Filaire et al., 2013; Nicolas et al., 2016). However, if the links between recovery-stress states and other markers of (mal)adaptation are well documented, the understanding of the process which underline the change from a state of well-being to a state of burnout and/or non-functional overreaching remain unclear (Carter et al., 2014; Eklund and Defreese, 2015).

Therefore, based on our results, it is a viable assumption that by building recovery strategies (e.g., focus on sleep quality, social relaxation, the feeling of success and personal accomplishment) coaches could reach to above athletes’ personal recovery mean. This should induce a positive cycle which would lead to higher levels of pleasant emotions (i.e., happiness, excitement) and lower levels of unpleasant ones (i.e., anxiety, dejection and anger). Consistent with the broaden-and-build theory (Fredrickson, 1998, 2001), the feeling of more pleasant emotions would then induce changes in thoughts, actions, and physiological responses (Fredrickson and Branigan, 2005) which could be of a particular interest during periods which lead to major competitions (i.e., tapering period).

Otherwise, athletes with higher levels of general stress and general recovery reported a greater decrease in excitement during development periods and a greater increase in excitement during tapering periods (i.e., general stress^∗^time^2^ and general recovery^∗^time^2^ were significant predictors of excitement). Furthermore, athletes with higher levels of sport-specific and total stress mentioned a more marked decrease in anxiety during the development periods, as well as a more marked increase during the tapering period than swimmers reporting lower scores of sport-specific and total stress (i.e., sport-specific stress^∗^time^2^ and total stress^∗^time^2^ were significant predictors of anxiety). Such results provide essential information for the management of development and tapering periods. One the one hand, it seems that during development periods (characterized by high training volumes and exhausting training loads), a good strategy would be to reduce general stress (e.g., social stress and general loads which are not dependent on the training process) in order to limit the decrease in the experience of excitement. On the other hand, during tapering periods, it could be a powerful strategy to adopt activities (e.g., social activities with friends and quality sleep) designed to increase general recovery and in turn promote the experience of excitement.

Practically, it seems that regularly monitoring the recovery-stress states of athletes is a particularly pertinent way to predict the levels (intercepts) and trajectories of emotional states in athletes. Due to the various implications of emotional experiences in sport settings (e.g., facilitating, debilitative or neutral) and their influence on performance and swimmers’ relationships (Hanin, 2012), it is a key issue for coaches and athletes to be able to anticipate risks of emotional disturbances during training periodization. By highlighting within-person synergies of a specific set of emotional states with perceived stress and recovery, this study therefore develops an operational and preventive approach for use by coaches and athletes in order to better target high-risk athletes. In view of the study results, a practical recommendation to coaches could be to carry out regular monitoring of their athletes’ stress-recovery states in order to obtain individual recovery and stress means for each specific training period. With this information, it may be possible to develop strategies in order to: (a) optimize the stress-recovery balance; (b) limit the negative effects of intensive training loads (i.e., during development periods); and (c) optimize the final step of the preparation (i.e., during tapering periods).

By using an individualized emotion scale, the SEQ offers the advantage of identifying a range of self-identified unpleasant and pleasant states which are relevant to athletes’ sport experience. However, our results are specific to the pleasant and unpleasant emotional states we measured, and precautions must be taken before broadening them to more general dimensions of emotional, mood and/or core affect states (Ekkekakis, 2012). Another limitation of the present study may be the fact that despite the *a priori* power-analysis, one’s may consider that the sample size we used is quite small. However, the aforementioned *a priori* power analysis we conducted provide evidence that the sample size is acceptable. Finally, the last limitation we identified refers to the importance of statistically assessing model-fit using chi-square testing across alternative models.

Future research should try to extend the results of this study by investigating operational strategies that optimize the stress-recovery balance. Such studies could be of particular interest in the fields of applied sport psychology and health psychology, because they will meet the need of coaches to manage the tapering periods, as well as those of athletes’ need to cope with exhausting training periods, which involve major risks of emotional disturbance and non-functional adaptations, especially in endurance sports like swimming.

## Conclusion

This study examined the trajectories of emotional states and their within-person synergies with perceived stress and recovery during a 4-month training period preceding the French swimming championships. Main results underline the central role played by perceived stress and recovery in the dynamics of emotional states experienced by high level swimmers during the period preceding the target competition of the season. It also provides support for the relevance of the SEQ in measuring emotions that swimmers experienced during a complete training periodization. Finally, this study gives some keys to coaches and athletes in detecting high-risk swimmers early, therefore enabling the adoption of early strategies to improve their recovery process and in turn their emotional states.

## Ethics Statement

The study protocol has received the approval from the University of Burgundy institutional research ethics board and by the swimming club’s executive committee. The swimmers’ coaches were contacted to obtain permission to approach their athletes about participation in the study. The athletes’ participation was voluntary and written informed consent was obtained from each individual prior to data collection (as well as from the parents of underage athletes).

## Author Contributions

PV carried out the data collecting. GM realized the statistical data processing. Finally, PV, GM, MN, and LM participated to the manuscript redaction.

## Conflict of Interest Statement

The authors declare that the research was conducted in the absence of any commercial or financial relationships that could be construed as a potential conflict of interest.
